# Supplementary material for: Vitamin D supplementation in Saudi Arabia: mothers’ knowledge, practices, and attitudes
Source: PeerJ. 2025 Aug 28;13:e19781. doi: 10.7717/peerj.19781 (PMC12399081; doi:10.7717/peerj.19781)
Supplement: Supplemental Information 1 [file peerj-13-19781-s001.pdf]

Vitamin D supplementation in Saudi Arabia: Mothers' perception - A cross sectional study

Research Tools

1. Demographic characteristics questionnaire

**1.Age**<br>Less than 25<br>≥25<30<br>≥30<35<br><35	**7.Your youngest child in months**<br>1-12<br>>12<24<br>24 and more
**2. Marital status**<br>Married<br>Divorced<br>Widower	**8.Living location**<br>Owing house<br>Rented house
**3.Educational level**<br>Uneducated<br>Primary<br>Average<br>Secondary<br>BA<br>Master's and above	**9.Monthly income**<br>Less than 5,000 riyals per month<br>From 5 - less than 10000 riyals per month<br>From 10-20 thousand riyals per month<br>More than 20 thousand riyals per month<br>together
**4. Functional status**<br>Employee<br>Unemployed	**10.How to breastfeed the youngest children**<br>Breast feeding<br>Bottle feeding<br>Both
**5.Nationality**<br>Saudi<br>Non-Saudi	
**6.Number of children**<br>One child<br>Two children<br>Three kids<br>Four children and more	

2. Vitamin D knowledge questionnaire

1.**Have you heard of Vitamin D?**		
Yes	1 point	1
No	0 points	
2.**Is there vitamin D in breast milk?**		
Yes	1 point	1
No	0 points	

I don't know	0 points	
3.**What is the source you learned about vitamin D? (You can choose more than one answer)**		
TV, radio, advertisements	1 point	7
Social media (Snapchat, Instagram, Facebook...)	1 point	
Friends – family	1 point	
Doctor/Nurse	1 point	
Magazines	1 point	
Books	1 point	
From studying at school/university	1 point	
Other		
4.**What are the benefits of vitamin D (you can choose more than one answer)**		
Bone health*	1 point	12
Boosts immunity*	1 point	
Helps a healthy pregnancy*	1 point	
Prevents stroke*	1 point	
Prevents diabetes*	1 point	
Prevents high blood pressure*	1 point	
Prevents arthritis*	1 point	
Prevent depression*	1 point	
Important for hair health*	1 point	
Prevents obesity and weight gain*	1 point	
Important for skin health*	1point	
Important for child health and development*	1 point	
I do not know	0 points	
5. **What are the sources of Vitamin D? (You can choose more than one answer) 3**		
The sun*	1 point	
The food*	1 point	
Food Supplements*	1 point	
I do not know	0 points	
6.**Where is Vitamin D found in food? (You can choose more than one answer)**		
Milk and dairy products*	1 point	4
Fruits	0 point	
Vegetables	0 points	
Fish*	1 point	
Yolk*	1 point	
Liver*	1 point	
I do not know	0 point	
		**Score 0-28**

3. Vitamin D practice related to your child questionnaire:

**1. I expose my child to the sun daily**		
Yes	1	
No	0	
**2. What part of the child's body exposed to the sun?**		
I do not expose my child to the sun	0	
Face only	1	

Hands only	1	
Feet and legs	2	
All the body*	3	
**3. Do you expose your child to the sun?**		
Directly*	1	
Indirectly (Behind the glass, for example)	0	
**4. What time do you expose your child to the sun?**		
I do not expose my child to the sun	0	
Before ten in the morning*	2	
After three in the afternoon	1	
Other times	1	
**5. What is the average number of hours your child is exposed to the sun per week?**		
I do not expose my child to the sun	0	
Less than an hour	1	
From one to two hours	2	
6 hours and more*	3	
**6. Do you know the appropriate dose for exposing your child to the sun?**		
Yes	1	
No	0	
**7. Do you give your child fortified doses of vitamin D (oral drops)?**		
Yes	1	
No	0	
**8. Commit to giving my child a daily fortified dose of vitamin D**		
Always*	3	
Sometimes	2	
Scarcely	1	
Do not apply	0	
**9. What are the reasons for not giving your child the supportive dose of vitamin D?**		
Forget	0	
Child's illness	0	
Because I expose it to the sun, there is no need to take vitamin D doses*	1	
Because the child gets a dose of vitamin D from formula or Cereal*	1	
Mother's preoccupation with other children	0	
Because the mother is busy with the job	0	
Because vitamin D doses are not necessary	0	
Other reasons (The child does not need vitamin D)	0	
		**0-28**

4. Vitamin D Attitudes questionnaire:

**1. Do you support taking vitamin D during pregnancy?**		
Yes	1	
No	0	
**2. Do you support taking vitamin D during breastfeeding?**		
Yes	1	

No	0	
**3. Do you support a blood test to detect the level of vitamin D in the body?**		
Yes	1	
No	0	
**4. If the answer to the previous question is yes, do you suggest that it be?**		
Periodically*	3	
From time to time	2	
At the request of the doctor	1	
**5. Do you support giving your child vitamin D since birth?**		
Yes	1	
No	0	
**6. Do you support exposing your child to the sun daily?**		
Yes	1	
No	0	
**7. Do you support the use of sunscreen while exposing your child to the sun?**		
Yes	1	
No	0	
		**0-12**

5. Practices towards taking vitamin D and exposure to sunlight

**1. I am currently taking vitamin D supplements**		
Yes	1	
No	0	
**2. I took vitamin D supplements during my pregnancy**		
Always	3	
Sometimes	2	
Scarcely	1	
never	0	
**3. I take vitamin D while breastfeeding**		
Always	3	
Sometimes	2	
Scarcely	1	
never	0	
**4. I was concerned that the food I prepared during the week contained vitamin D**		
Always	3	
Sometimes	2	
Scarcely	1	
Permanently	0	
**5. I exposed to the sun while out of the house**		
Yes	1	
No	0	
**6. I make sure that the food I eat during the week contains vitamin D**		
Yes	2	
Sometimes	1	
No	0	
**7. I make sure to be exposed to the sun daily**		
Yes	1	
No	0	
**8. How many hours per week are you exposed to the sun?**		
less than an hour	0	

From one to two hours	1	
6 hours and more*	2	
**9. What parts of the body are exposed to the sun?**		
Face	1	
Hands	1	
Face and hands	2	
Face, hands, and legs*	3	
**10. What is the right time to be exposed to the sun?**		
Before ten in the morning	2	
From ten to three in the evening	1	
After three o'clock in the evening	1	
**11. Do you use sunscreen when you go out in the sun?**		
Yes	1	
Sometimes	0	
No	0	
**12. How often do you use sunscreen while out in the sun?**		
Always	3	
Sometimes	2	
Scarcely	1	
Permanently	3	
**13. Is there a conflict between applying sunscreen and exposure to the sun?**		
Yes	1	
No	0	
I do not know	0	
		**0-26**

The total score can be calculated by summing the points for each question. Higher scores indicate better practices towards vitamin D supplementation and sun exposure.
