# Supplementary material for: Vitamin D supplementation in Saudi Arabia: mothers’ knowledge, practices, and attitudes
Source: PeerJ. 2025 Aug 28;13:e19781. doi: 10.7717/peerj.19781 (PMC12399081; doi:10.7717/peerj.19781)
Supplement: Supplemental Information 2 [file peerj-13-19781-s002.pdf]

استبيان قياس معلومات وموقف الامهات حول فيتامين د لاطفالهن في المراكز الصحية الاولية

الجزء الاول : المعلومات الشخصية للامهات :

1.العمر :

أقل من 25   25 إلى أقل من 35   أكثر من 35

2. عدد الاطفال لديك :

1 ، 2 ، 3 ، 4 وأكثر

3. عمر أصغر طفل :

1-12 شهر ، 12-24 شهر ، 24-36شهر ، 36-48 شهر

4. المستوى التعليمي :

أمية ، ابندائي ، متوسط ، ثانوي ، جامعي

5. الحالة الوظيفية :

أعمل ، لا أعمل

الجزء الثاني : معلومات الامهات بفيتامين د

1. **هل سمعت بفيتامين د ؟** نعم ، لا

2. **ما هو المصدر الذي علمت منع بفيتامين د ؟**

الوسائل الاجتماعية (كالتلفاز ، الردادیو ، النت) ، الاصدقاء ، العائلة ، الطبيب

3. **ماهي فوائد فيتامين د؟ اختاري مما يلي**

صحة للعظام ، يعزز المناعة ، يساعد على الحمل الصحي ، يمنع الجلطة الدماغية ، منع مرض السكري منع ارتفاع ضغط الدم ، منع التهاب المفاصل ، منع الاكتئاب ، مهم لصحة الشعر ، يمنع السمنة وزيادة الوزن ، مهم لصحة الجلد ، مهم لصحة الطفل ونموه ، لا أعرف

4. ماهي مصادر فيتامين د المتوفرة ؟

الشمس ، الطعام ، جرعات دوائية ، لا أعرف

5. أين يوجد فيتامين د في الطعام ؟ اختاري مما يلي

الفواكه ، الخضار ، السمك ، البيض ، صفار البيض ، الخضار الورقية

الجزء الثالث : ممارسة الأمهات تجاه أطفالهن فيما يتعلق بفيتامين د ومكملاته

1. **أعرض طفلي للشمس بشمل يومي ؟** نعم ، لا

2. **أي جزء من جسم الطفل تعرضيه للشمس ؟**

الوجه ، اليدين ، القدمين ، كل الجسم

3. **أي وقت تعرضين طفلك للشمس ؟**

قبل العشرة صباحا

من العاشرة الى الثلاثة مساء

بعد الثالثة مساء

4. **ما هو معدل الساعات الاسبوعية التي يتعرض فيها طفلك للشمس ؟**

أقل من ساعة

من ساعة الى ساعتين

6 ساعات وأكثر

5. **هل تعطين طفلك جرعات مدعمة من فيتامين د ؟ نعم لا**

6. **التزم بإعطاء الجرعة اليومية المدعمة من فيتامين د لطفلي ؟**

دائما ، أحيانا ، نادرا

7. **ما هي أسباب عدم التزامك بإعطاء الحرعة الداعمة من فيتامين د لطفلك ؟**

أنسى ، مرض الطفل ، غير مهم ، لأني أعرضه للشمس فلا داعي ، الطبيب

الجزء الرابع : موقف الامهات وتجاه فيتامين د ومكملاته :

1. هل توافقين على أهمية أخذ فيتامين د خلال فترة الحمل ؟ نعم ، لا
2. هل توافقين على أهمية أخذ فيتامين د خلال ففترة الرضاعة ؟ نعم ، لا
3. هل توافقين على أهمية الكشف عن مستوى فيتامين د بالجسم ؟ نعم لا
4. هل توافقين على أهمية إعطاء طفلك فيتامين د منذ ولادته ؟ نعم لا
5. هل توافقين على أهمية تعريض طفلك للشمس بشكل يومي ؟ نعم لا

الجزء الخامس : ممارسة الأمهات تجاه تناول فيتامين د والتعرض لأشعة الشمس

1. **أتناول مكملات فيتامين د حاليا ؟** نعم لا
2. **تناولت مكملات فيتامين د أثناء حملي الاخير ؟** دائما ، أحيانا ، لا
3. **أتناول فيتامين د أثناء الرضاعة ؟** نعم ، أحيانا ، لا
4. **أهتم بأن يحتوي الطعام الذي أتنتوله خلال الاسبوع أن يحتوي غلى فيتامين د ؟** نعم ، أحيانا ،لا
5. **أحرص على التعرض للشمس يوميا ؟** نعم لا
6. **ماهو معدل الساعات الاسبوعية التي تتعرضين فيها للشمس ؟**

أقل من ساعة ، من ساعو الى ساعتين ، 6 ساعات وأكثر

7. **ماهو الوقت الناسب لتعرض لاشعة الشمس ؟**

قبل العشرة صباحا ، من العاشرة الى الثالثة مساء ، بعد الساعة الثالثة

8. هل تستخدمين واقي شمس عند الخروج ؟ نعم ، أحيانا ، لا
